# Hybrid EEG—Eye Tracker: Automatic Identification and Removal of Eye Movement and Blink Artifacts from Electroencephalographic Signal

**DOI:** 10.3390/s16020241

**Published:** 2016-02-19

**Authors:** Malik M. Naeem Mannan, Shinjung Kim, Myung Yung Jeong, M. Ahmad Kamran

**Affiliations:** Department of Cogno-Mechatronics Engineering, Pusan National University, 2 Busandaehak-ro 63beon-gil Geumjeong-gu, Busan 609-735, Korea; naeem@pusan.ac.kr (M.M.N.M.); shinjungkim@pusan.ac.kr (S.K.); malik@pusan.ac.kr (M.A.K.)

**Keywords:** electroencephalogram, eye tracker, ocular artifacts, independent component analysis, auto-regressive exogenous model, affine projection algorithm, composite multi-scale entropy, median absolute deviation

## Abstract

Contamination of eye movement and blink artifacts in Electroencephalogram (EEG) recording makes the analysis of EEG data more difficult and could result in mislead findings. Efficient removal of these artifacts from EEG data is an essential step in improving classification accuracy to develop the brain-computer interface (BCI). In this paper, we proposed an automatic framework based on independent component analysis (ICA) and system identification to identify and remove ocular artifacts from EEG data by using hybrid EEG and eye tracker system. The performance of the proposed algorithm is illustrated using experimental and standard EEG datasets. The proposed algorithm not only removes the ocular artifacts from artifactual zone but also preserves the neuronal activity related EEG signals in non-artifactual zone. The comparison with the two state-of-the-art techniques namely ADJUST based ICA and REGICA reveals the significant improved performance of the proposed algorithm for removing eye movement and blink artifacts from EEG data. Additionally, results demonstrate that the proposed algorithm can achieve lower relative error and higher mutual information values between corrected EEG and artifact-free EEG data.

## 1. Introduction

In recent years, non-invasive neuro-imaging has become a valuable research tool to understand the underlying functionality of the brain [1,2,3,4,5]. Electroencephalogram (EEG) with the advantages of portability and high temporal resolution is a non-invasive brain-imaging technique used to measure different physiological states of the brain with amplitude typically in order of a few microvolts [6,7]. Unfortunately, measurements from EEG are highly contaminated with eye movement and blink artifacts which are several times higher in magnitude as compared to neuronal activity [8,9,10,11,12,13]. This issue has become a recurrent problem, for example in brain-computer interface (BCI) where it has been proved to decrease the classification accuracy [14].

Various methods to tackle this challenging task have been proposed in the literature. A straight forward way to reduce ocular artifacts is to discard the artifactual epochs from EEG data. However, this may cause a considerable data loss related to neuronal activity and it also requires a huge amount of time. In contrast, several automated methods have been proposed to detect and remove/reduce ocular artifacts in past. These methods can be divided into two main categories, *i.e.*, regression based methods [9,15,16,17,18,19,20] and blind source separation techniques [21,22,23,24,25,26,27].

**Figure 1:**
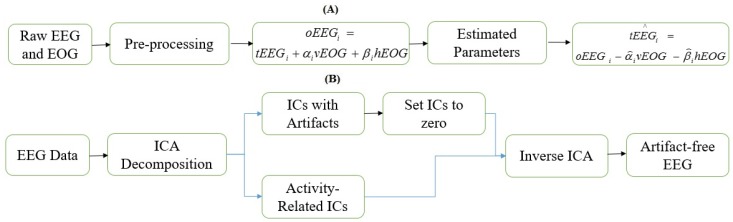
Schematic diagrams. (**A**) Regression method; (**B**) Independent component analysis.

In regression algorithms, propagation coefficients of ocular artifacts are calculated to estimate the amount of electrooculography (EOG) signal present in EEG signal and subsequently the estimated EOG is subtracted from EEG to obtain artifact-free EEG [28,29,30,31,32]. Since EOG also captures the neuronal activity from prefrontal cortex, therefore the common neuronal activity in EEG and EOG might be lost in regression methods and due to this bidirectional contamination problem these algorithms were proved to be less effective [29]. Figure 1A shows the schematic diagram of regression algorithms.

On the other hand, the second class of methods are based on the assumption that the neuronal activity and artifactual activity are independent from each other. Most commonly used method in blind source separation is independent component analysis (ICA) [21,31,32]. In ICA based algorithms, EEG signal is decomposed into several independent components (ICs), which are then classified into neuronal-activity-related and artifactual ICs. The selected artifactual ICs are then removed to obtain artifact-free EEG. The main issue of this methodology is the selection of artifactual components, usually this can be done by the visual inspection of an expert but this approach might lead to misclassification of ICs and divergent results [33]. Recently, several automated criteria have been developed to tackle this issue [26,32,33,34] and they showed significant improvement in terms of artifact removal from EEG data but the distortion produced due the removal of those ICs in EEG signal was left unaddressed, since an artifactual IC may contain neuronal activity along with artifacts [35]. The schematic diagram illustrating the ICA based removal of ocular artifacts is shown in Figure 1B.

Recently, researchers proposed to combine these two methodologies to remove ocular artifacts from EEG by utilizing their advantageous features [29,36]. Although these methods proved to be effective in removing ocular artifacts but they always require simultaneous EOG recordings, which is not plausible in applications like BCI. To this extent, Kierkels and coauthors [37] proposed to use an eye tracker for eye movement artifact removal from EEG. They used eye tracker to generate eye positions, which were used as inputs to Kalman filter to remove eye movement artifacts. Although, their method achieved improved results over other artifact removal techniques but it is not able to deal with blink artifacts. Later, Noureddin and colleagues [38] used a high speed eye tracker to propose a regression based technique with recursive least square and H^∞^ filters. Plöchl and co-authors [33] proposed a simple criteria for identifying artifactual ICs based on the events of eye tracker and they replaced the selected ICs with zero to get clean EEG.

**Figure 2:**
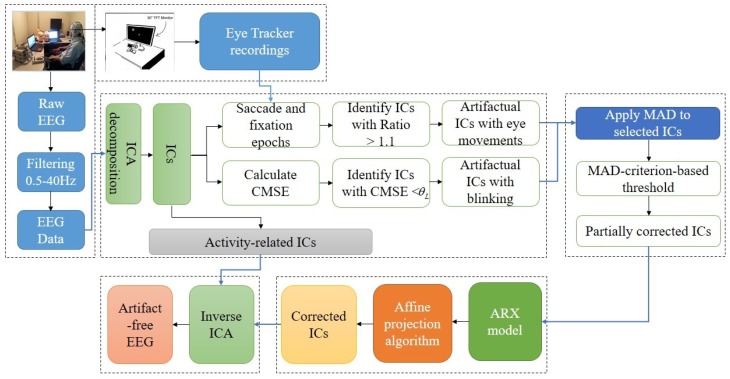
Schematic diagram of the proposed algorithm.

In this paper, hybrid EEG and eye tracker system is used to develop a novel adaptive framework which automatically detects and removes eye movement and blink artifacts from EEG data. In contrast to the EOG based algorithms, the proposed algorithm is developed such that eye tracker and frontal EEG electrodes are used to detect and remove ocular artifacts. The proposed methodology combines the advantageous features of ICA and system identification to remove eye movement and blink artifacts. The first step of the proposed algorithm is to obtain ICs by ICA decomposition of EEG data. In the next step, we proposed to use composite multi-scale entropy and eye tracker events to automatically identify blink and eye movement artifacts related ICs, respectively. These artifactual ICs are filtered with median absolute deviation to remove high magnitude ocular activities and then processed to an auto-regressive exogenous model to completely remove eye movement and blink artifacts. The parameters of the auto-regressive exogenous model are estimated using affine projection algorithm. In the final step, inverse ICA is used to reconstruct EEG signal by back projecting all ICs. The performance of the proposed algorithm is demonstrated through results on experimental and standard EEG datasets. Furthermore, the proposed algorithm is compared with two state-of-the-art techniques namely ADJUST based ICA [34] and REGICA [30]. Relative error and mutual information are used as an evaluation indexes to measure the ability of removing ocular artifacts by the proposed algorithm. Paired *t*-test is used to validate the significant improvement in removing ocular artifacts by the proposed algorithm over previous methods. Results show that the proposed method is efficient for automatic detection and removal of eye movement and blink artifacts from EEG signals. The schematic diagram and summary of the proposed algorithm are shown in Figure 2 and Table 1, respectively.

The remainder of this paper is organized as follows. In Section 2, a detailed description of the datasets and proposed algorithm is presented. Evaluation indexes used in this study are formulated in Section 3 followed by the results and discussion sections. Finally, the main idea and conclusions are summarized in last section. 

## 2. Materials and Methods

### 2.1. Materials

In this paper, we used experimental and standard EEG datasets to demonstrate the performance of the proposed algorithm.

#### 2.1.1. Participants

EEG and eye tracker measurements were acquired from 5 healthy subjects, all male, mean age 28. Experiment was conducted under the Declaration of Helsinki. The experiment was approved by the Institutional Review Board of Pusan National University. The experiment was conducted in a confined room with dim light to avoid environmental disturbance.

#### 2.1.2. Experimental Procedure

All the participants were seated in an armchair at a distance of about 1m from a monitor screen (Samsung, SyncMaster B1940, 19”). The participants were asked to perform a task involving different eye movements and blinks. The experiment starts with the blank screen for 5 s. During this task, a red dot appears on the screen for the duration of 2 s at nine different positions in a square region of 960 × 960 pixels. All the participants were instructed to follow the moving dot. Before every position change of the dot, a fixation cross appeared on the screen for 1 s, thus providing the cue for the subject to follow the dot. Each participant was instructed to blink when “**blink**” appears on the screen. A blank screen appears for 5 s after every blink. All participants performed three experimental blocks. Each experimental block consisted of 18 saccade trials and 18 fixation trials.

#### 2.1.3. EEG Recordings

**Figure 3:**
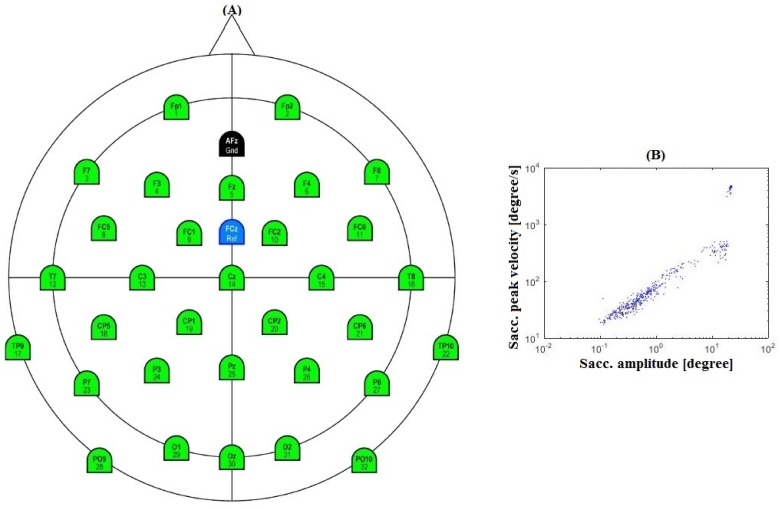
(**A**) Electroencephalogram (EEG) electrode configuration; (**B**) Distribution of saccade amplitude.

EEG data were recorded using BrainAmp DC amplifier with an ActiCap 32-channel active electrode system developed by Brain Products GmbH, Gilching, Germany. All the data were sampled at a rate of 500 Hz. All the electrodes were placed according to international 10–20 system as shown in Figure 3A. The impedance of all the electrodes were reduced below 5 kΩ. 

#### 2.1.4. Eye Tracker Recordings

The eye movements were recorded with a video eye tracking system Eyelink 1000 developed by SR research Ltd., Ottawa, ON, Canada. The sampling rate was 1000 Hz. The velocity threshold of 30 °/s was used to define saccades, an acceleration threshold of 8000 °/s^2^ and a minimum deflection threshold of 0.1°. Figure 3B shows the distribution of saccades amplitude.

#### 2.1.5. Preprocessing

In order to synchronize EEG and eye tracker, eye tracker data was down-sampled at the sampling rate of 500 Hz. EEG and eye tracker data were then aligned by cutting them into trials according to the triggers that were simultaneously sent to both, the EEG and the eye tracking system [32]. The EEG data was band pass filtered between 0.5–40 Hz. All the processing and analysis were done in Matlab (Mathworks) and EEGLAB using Intel core i3, 2.4 GHz with 4 GB RAM laptop.

#### 2.1.6. Standard Dataset

The principle measure to evaluate the performance of the proposed algorithm is to check its ability of removing artifactual activities from standard dataset. Preparatory to such an evaluation, publicly available contaminated EEG dataset with eye tracker signals was utilized [39]. In this experiment, participant read lists of five words from left to right. Their task was to report whether the list contained the name of an animal. Eye movements were recorded binocularly with an Eyelink 1000 tracker at 1000 Hz.

EEG data was recorded from 72 channels with Biosemi Active amplifiers at a rate of 512 Hz. In the preprocessing part, EEG data was filtered between 0.5 and 40 Hz and the baseline was removed from all of the data.

### 2.2. Methods

#### 2.2.1. Independent Component Analysis

ICA is a statistical technique used for decomposing multichannel data into several ICs under the following assumptions [40]:

1. The number of ICs are less than or equal to the number of observed signals.
2. The artifactual and cerebral sources are linearly mixed and statistically independent.
3. Propagation delays through the missing medium (brain) are negligible.

The basic purpose of ICA is to consider the non-Gaussianity of the measured signal and to find their projections. Mathematical mode of ICA, for the observed EEG data can be represented as

(1) x(k)=As(k),k=1, 2, 3, ... , N

where $x(k)\in {ℜ}^{M\times 1}$ is the measured EEG signal, $s(k)\in {ℜ}^{M\times 1}$ is the corresponding IC, $A\in {ℜ}^{M\times M}$ is the full rank unknown mixing matrix, *k* is discrete time, *N* is the number of samples and *M* is the number of ICs. Since the total number of ICs contributing to EEG data are unknown, therefore in this study they are supposed to be equal to the number of electrodes used in EEG data acquisition. Given x(k), the issue is how to estimate both A and s(k). The ICs ${\hat{s}}_{i}(k),\;i=1,\;2,\;3,\;...,\;M$ can be represented as

(2) $${\hat{s}}_{i}(k)=w_{i}^{T}x(k),\;k=1,\;2,\;3,\;...\;,\;N$$

where $w_{i}$ is a column vector. After estimation of each $w_{i}$, the ICs can be obtained by using the following expression

(3) $$\hat{s}(k)=Wx(k),\;W\approx A^{-1}$$

The algorithm implemented in this paper is infomax ICA with default parameters using *runica* function of the EEGLAB tool box (MATLAB, Torrance, CA, USA) [41]. These parameters involved pre-sphering of the data and to avoid training if weight change was less then 10^−6^.

#### 2.2.2. Features Computation

##### Eye Blinks

Entropy has been found to be very useful in detecting artifactual components in physiological signal. Costa and coauthors [42] developed a multi-scale methodology to calculate the entropy of the biological signal and their method has been proved to be effective then Shannon’s and Renyi’s entropy [43]. In the light of the above, we proposed to use composite multi-scale entropy [44] to automatically identify blink related artifactual ICs.

The composite multi-scale entropy rationale is that the blink components have low entropy values as compared with neural components, because the pattern of blink activity is more regular than the neuronal activity detected in EEG signals. Hence the utility and value of composite multi-scale entropy as a statistical tool for identification of blink related ICs. The step-wise procedure for computation of composite multi-scale entropy is as follows:

(1) Let $u_{i}$be the ith IC, the *l*th coarse-grained time series for a scale factor of τ, $z_{l}^{\left(\tau \right)}=\left\{z_{l,1}^{\left(\tau \right)}\;z_{l,2}^{\left(\tau \right)}\;\cdots \;z_{l,p}^{\left(\tau \right)}\right\}$ can be defined as
  (4) $$z_{l,j}^{\left(\tau \right)}=\frac{1}{\tau }\sum_{i=(j-1)\tau +l}^{\tau +l-1}u_{i},\;1\leq j\leq \frac{N}{\tau },\;1\leq l\leq \tau$$
(2) In the composite multi-scale entropy algorithm, at a scale factor of τ, the sample entropies (SampEns) of all coarse-grained time series are calculated and the composite multi-scale entropy value is defined as the mean of τ entropy values. That is
  (5) $$CMSE(u,\tau ,m,r)=\frac{1}{\tau }\sum_{l=1}^{\tau }SampEn(z_{l}^{\left(\tau \right)},m,r)$$
  where *CMSE* represents the composite multi-scale entropy. In this study, the composite multi-scale entropy was calculated from τ=1 to 20, and the sample entropy of each coarse-grained IC was calculated with *m* = 2 and r=0.15σ, where σ is the standard deviation of the IC [42,44].

Eye blinks typically generate abrupt amplitude jumps in frontal electrodes. As blink activities are notably different from neuronal activities, it is possible to detect them using a suitable threshold for composite multi-scale entropy. Since the composite multi-scale entropy values for ocular activities are expected to be low, in the proposed adaptive algorithm, the threshold for identifying blink related ICs is defined as,

(6) $$\theta_{L}=\bar{x}-1.64s$$

where $\theta_{L}$ represents the threshold, $\bar{x}$ and *s* are the mean and standard deviation of the composite multi-scale entropy values for all ICs. All ICs with composite multi-scale entropy values above the threshold are assumed to be neuronal-activity-related ICs, while the others are selected for reconstruction.

##### Horizontal Eye Movements

Horizontal eye movements generate large amplitude fluctuations in frontal channels that are typically slower than those of blinks, therefore not efficiently identifiable by the composite multi-scale entropy. To identify horizontal eye movement related artifacts, all the ICs were portioned into saccade and fixation epochs [33]. Saccade epochs were defined as the time between horizontal eye movements start and end as given by the eye tracker. An additional interval of 5 ms before and 10 ms after was added to saccade epochs. In contrast, the fixation epochs were defined as the time between saccade epochs. Finally, the ratio of the mean variance for saccade and fixation epochs was calculated for all the ICs:

(7) $$Ratio=\frac{mean(\text{var }iance_{saccade})}{mean(\text{var }iance_{fixation})}$$

If for given IC the ratio of mean variance defined in Equation (7) was greater than 1.1, the corresponding IC was selected as artifact related IC and subsequently processed for correction.

##### Vertical Eye Movements

Since the time course of artifacts caused by vertical eye movements is similar to the one generated by horizontal eye movements, the feature described in Equation (7) can be used to identify vertical eye movements related ICs.

#### 2.2.3. Median Absolute Deviation

Once the artifactual ICs are identified, they are then processed for correction by a two-step methodology. In the first step, the ocular activities (outliers) that are set to zero are only those notable ones that are of a considerably high magnitude. In this way, the amount of neuronal activity in the ocular artifacts related components can be retained. In the present study, median absolute deviation was used to detect and remove high-magnitude ocular activities from the components [45]. The step-wise procedure for such removal is as follows:

(1) Evaluate the median absolute deviation of the identified ocular activity among the identified artifactual ICs (median absolute deviation is defined as the median of the absolute deviation from the median)
  (8) $$MAD=bM\left(\left|u_{i}(k)-M(u_{i})\right|\right)$$
  where MAD is the median absolute deviation, M is the median, $M(u_{i})$ is the median of the *i*th artifactual IC, b is a constant;
(2) If $u_{i}(j)$ exceeds the criteria calculated using Equation (9), it is thresholded to zero:
  (9) $$M(u_{i})-3*MAD<u_{i}(k)<M(u_{i})+3*MAD$$
  (10) $$\frac{u_{i}(k)-M(u_{i})}{MAD}>\left|\pm 3\right|$$

#### 2.2.4. Auto-Regressive Exogenous Model

The procedure described above will only remove those artifactual components which can be clearly seen and detectable in ocular artifacts related ICs. Auto-regressive exogenous model is used to completely remove ocular artifacts from identified ICs. It is expected that the amount of neuronal activity included in identified components is much lower than that of present in contaminated EEG. Therefore, in the present study we applied auto-regressive exogenous model to ocular artifacts related ICs instead of EEG data. A linear auto-regressive exogenous model can be used to remove ocular artifacts from artifactual ICs by the following equation

(11) $$y(k)+\sum_{i=1}^{p}a_{i}y(k-i)=\sum_{j=1}^{q}b_{j}E_{Fp1}(k-j)+\sum_{l=1}^{r}c_{l}E_{Fp2}(k-l)+e(k)$$

where *y* is the output, $E_{Fp1}$ and $E_{Fp2}$ are the inputs of the auto-regressive exogenous model representing Fp1 and Fp2 electrode of EEG at discrete time *k*, $a_{i}$, $b_{j}$ and $c_{l}$ are the parameters to be estimated, *p*, *q* and *r* represent the order of the model and e(k) is the error assumed to be white-noise disturbance. Mathematically, Equation (11) can be rewritten in linear regression as below

(12) $$y(k)=X^{T}(k)\beta +e(k)$$

where X and β have the following form

(13) $$\begin{array}{l} X(k)={\left[-y(k-1),\cdots ,-y(k-p),E_{Fp1}(k-1),\cdots ,E_{Fp1}(k-q),E_{Fp2}(k-r),\cdots ,E_{Fp2}(k-r)\right]}^{T}\\ \beta ={\left[a_{1},\cdots ,a_{p},b_{1},\cdots ,b_{q},c_{1},\cdots ,c_{r}\right]}^{T} \end{array}\}$$

#### 2.2.5. Affine Projection Algorithm

The error signal can be obtained by subtracting the estimated output from the desired signal. Mathematically, it can be written as [46]

(14) $$e(k)=y(k)-X^{T}(k)\hat{\beta }(k-1)$$

Then, the objective of the estimation problem is to minimize the squared Euclidean norm of

(15) $${\left\|\hat{\beta }(k+1)-\hat{\beta }(k)\right\|}_{2}^{2}$$

Subject to constraints

(16) $$y(k)-X^{T}(k)\hat{\beta }(k)=0$$

Thus, an update equation is required such that the difference between two consecutive estimations of the unknown parameters is minimized. This can be achieved by using the method of Lagrange multipliers which converts the constrained minimization into an unconstrained one. Thus, the cost function can be defined as

(17) $$J(k)={\left\|\hat{\beta }(k)-\hat{\beta }(k-1)\right\|}_{2}^{2}+\left[y(k)-X^{T}(k)\hat{\beta }(k)\right]\theta (k)$$

where θ is the Lagrange multipliers vector. Taking the gradient of *J*(*k*) with respect to $\hat{\beta }(k)$ and equating the result to zero, we can find

(18) $$\hat{\beta }(k)=\hat{\beta }(k-1)+\frac{1}{2}X(k)\theta (k)$$

Using Equations (12) and (14) and the Lagrange method, we can write

(19) $$\theta (k)=2{\left[X^{T}(k)X(k)\right]}^{-1}e(k)$$

Substituting Equation (19) into Equation (18), the optimal change in the parameter vector can be written as

(20) $$\hat{\beta }(k)=\hat{\beta }(k-1)+X(k){\left[X^{T}(k)X(k)\right]}^{-1}e(k)$$

A step size parameter μ and regularization constant δ can be used to modify the above equation for efficient updating of the parameter vector as

(21) $$\hat{\beta }(k)=\hat{\beta }(k-1)+\mu X(k){\left[\delta I+X^{T}(k)X(k)\right]}^{-1}e(k)$$

## 3. Evaluation Index

Since it is not possible to exactly evaluate the performance of proposed methodology that how much artifacts from EEG data has been removed due to the unknown contributions of the neuronal activity and ocular activity. That is, it is not possible to measure signal to artifact ratio. But the performance of the proposed algorithm can be evaluated using data in the intervals before artifact contamination [47]. To test how well the proposed algorithm performed in comparison to the conventional methods, we asked an independent EEG expert to tag all intervals in our data that he considered as ocular artifacts related. The selection was done by visual inspection of the EEG time series. All the remaining data was considered as artifact-free EEG data and used to calculate the performance metrics. In this study, we used two performance measures to quantitatively verify the ability of the proposed algorithm in removing ocular artifacts and how much the EEG signals are distorted after the artifact rejection procedure.

### 3.1. Relative Error

In the present study, the relative error criteria was used to evaluate the proposed algorithm’s utility in removing ocular artifacts from EEG signals and comparing it with the results of the conventional methodologies. Relative error is defined as [47]

(22) $$RE=\frac{\left|EEG_{free}-EEG_{out}\right|}{\left|EEG_{free}\right|}$$

where RE represents the relative error, $EEG_{free}$ is the artifact-free EEG signal as selected by EEG expert, $EEG_{out}$ is the artifact corrected EEG signal from the proposed algorithm and | ⋅ | denotes the norm calculation for vector.

### 3.2. Mutual Information

The mutual information index was calculated to measure the mutual dependence of the artifact-free EEG signal and output EEG from the proposed method. Mathematically, it is found by using Kullback-Leibler divergence between the probability distribution function as [24]

(23) $$MI=\int_{-\infty }^{\infty }\;\int_{-\infty }^{\infty }f(a,b)\log\frac{f(a,b)}{f(a)f(b)}dadb$$

where MI is the mutual information, f(a,b) is the joint pdf and f(a) and f(b) are the marginal pdfs. Mutual information is calculated using an open source MATLAB function minfo.m developed by Dr. Jason Palmer [48]. If the mutual information between artifact-free EEG and output EEG from proposed method is large, it means they are closely related.

## 4. Results

This paper presents an automatic framework based on ICA and auto-regressive exogenous model to identify and remove ocular artifacts from EEG signals by combining EEG and eye tracker. The effectiveness of the proposed algorithm was demonstrated using experimental and standard EEG datasets. The performance of the proposed algorithm is compared with two conventional methods, *i.e.*, ICA and REGICA to verify the significant improvement of results. In this study, ADJUST as implemented in EEGLAB toolbox is used to represent ICA based algorithms and REGICA is used to represent methods based on the combination of ICA and regression.

**Figure 4:**
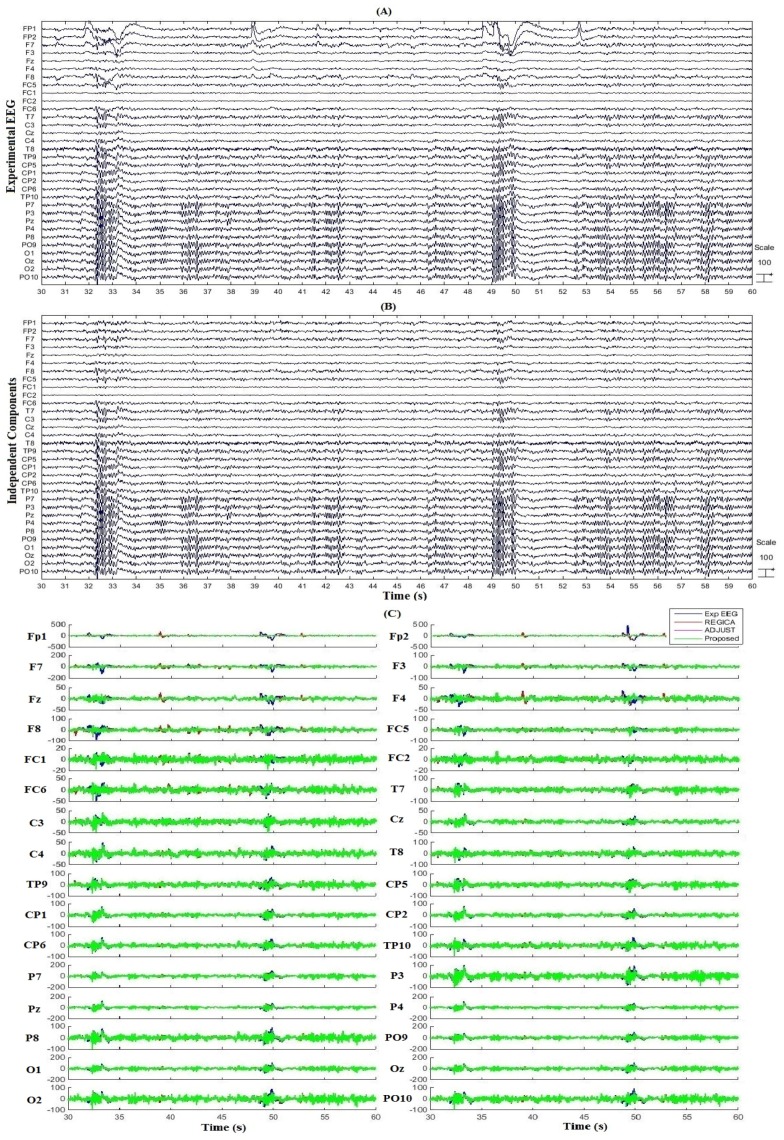
Results on experimental dataset. (**A**) Experimental EEG data for one subject; (**B**) Independent components (ICs) obtained from independent component analysis (ICA) decomposition of EEG data; (**C**) Comparison of the corrected EEG by the proposed algorithm and conventional algorithms.

Five experimental EEG datasets were used to verify the performance of the proposed algorithm. Figure 4 plots the results of artifact removal by the proposed algorithm for one subject. Figure 4A shows the experimental EEG data for one subject, Figure 4B, the corresponding ICs obtained from ICA decomposition of the EEG data, and Figure 4C, comparison of the artifact-free EEG data obtained after implementation of the proposed algorithm and conventional algorithms. In Figure 4B, ICs 2 and 13 are blink related components and ICs 9 and 23 are eye movement related components as identified by the proposed algorithm. It is evident in Figure 4C that the ocular artifacts were significantly removed by the proposed algorithm, in contrast to Figure 4A. Furthermore, the comparison with the conventional algorithms show the improved performance of the proposed algorithm.

**Figure 5:**
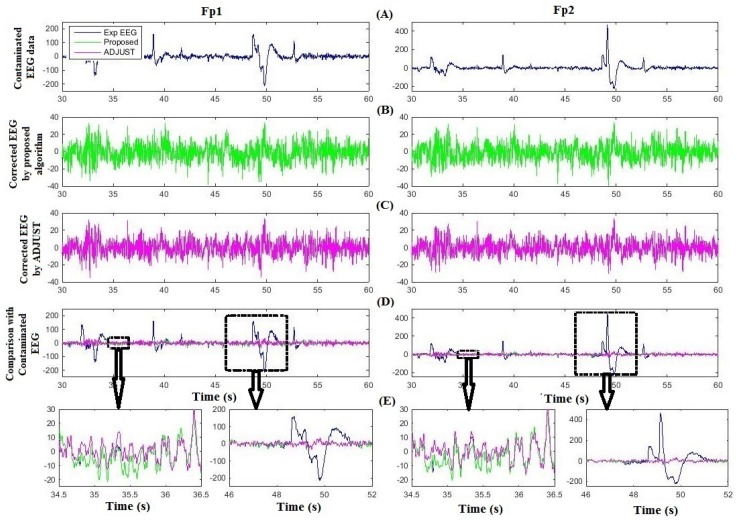
Comparison of the proposed algorithm with ADJUST using experimental data. (**A**) Contaminated experimental EEG data at Fp1 and Fp2; (**B**) Corrected EEG by the proposed algorithm; (**C**) Corrected EEG by ADJUST; (**D**) Comparison of corrected EEG with contaminated experimental EEG. (**E**) Partial enlargement of highlighted regions.

**Figure 6:**
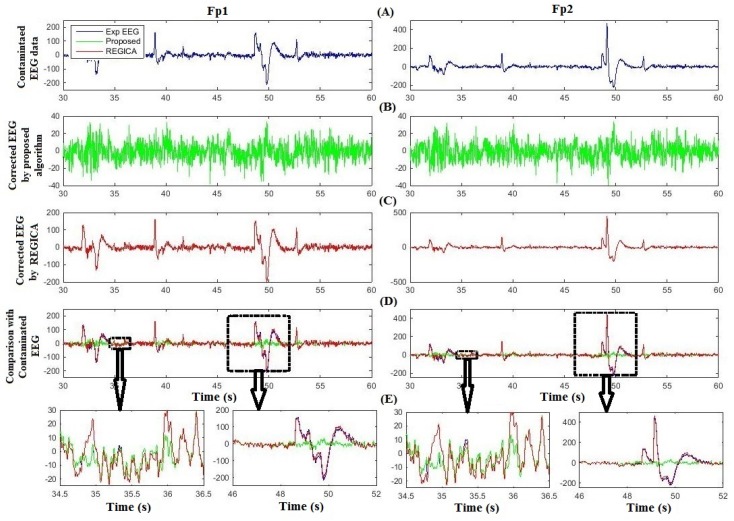
Comparison of the proposed algorithm with REGICA using experimental data. (**A**) Contaminated experimental EEG data at Fp1 and Fp2; (**B**) Corrected EEG by the proposed algorithm; (**C**) Corrected EEG by REGICA; (**D**) Comparison of corrected EEG with contaminated experimental EEG; (**E**) Partial enlargement of highlighted regions.

Figure 5 compares the proposed method and ADJUST for artifact removal for one subject at Fp1 and Fp2. Figure 5A shows the contaminated EEG data; Figure 5B,C are the corresponding artifact-free EEG after implementation of the proposed algorithm and ADJUST, respectively; Figure 5D compares the proposed algorithm and ADJUST with the contaminated EEG data. The two black boxes on the left and two on the right in Figure 5D highlight the uncontaminated and contaminated regions of the EEG signals at Fp1 and Fp2, respectively, which are partially enlarged in Figure 5E. Figure 5E shows that ADJUST causes distortion and loss of neuronal activity from the EEG data, whereas the proposed algorithm successfully preserves the neuronal-activity-related EEG signal intact. Indeed, in Figure 5E, it can be seen that the proposed algorithm performs better in removing ocular artifacts and reconstructing the EEG signal. Figure 6 illustrates the comparison of corrected EEG by proposed method with REGICA method. Figure 6D, meanwhile, compares the proposed algorithm with the REGICA at Fp1 and Fp2, respectively, and indicates that the proposed algorithm offers significantly better performance (Figure 6E).

**Figure 7:**
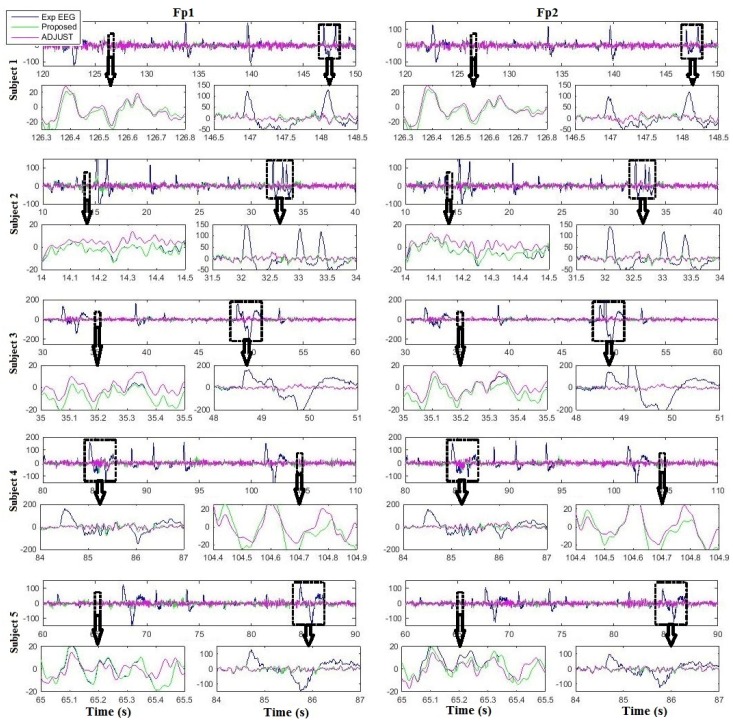
Comparison results of the proposed algorithm and ADJUST for all subjects at Fp1 and Fp2.

**Figure 8:**
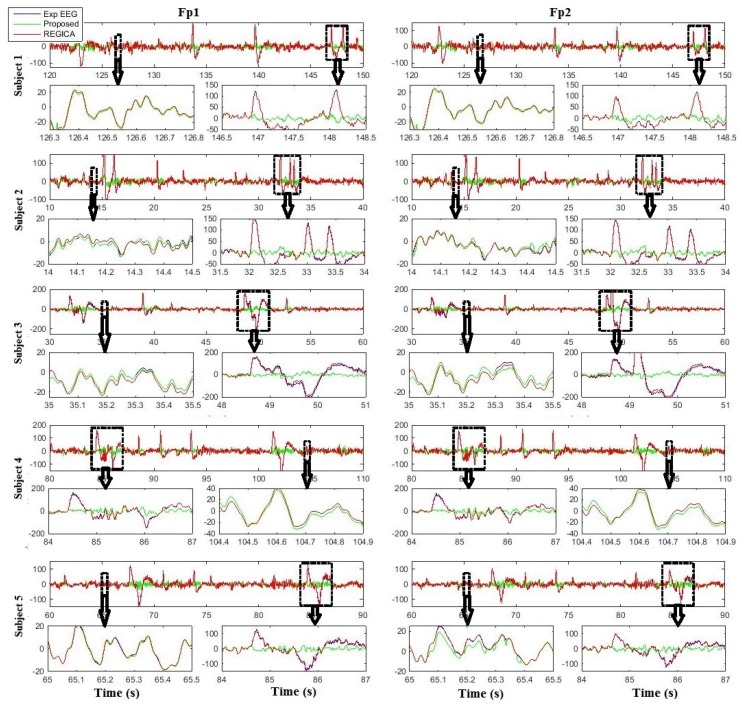
Comparison results of the proposed algorithm and REGICA for all subjects at Fp1 and Fp2.

Figure 7 and Figure 8 show the comparison results of the proposed algorithm with ADJUST and REGICA for all subjects at Fp1 and Fp2, respectively. It can be seen that the proposed algorithm outperforms conventional methods in removing ocular artifacts from EEG data as well as in preserving the neuronal activity related EEG signal (enlarged panels). In order to investigate the effect of the different ocular artifacts reduction methods on artifact-free data in frequency domain, the power spectral density (PSD) is computed and compared.

**Figure 9:**
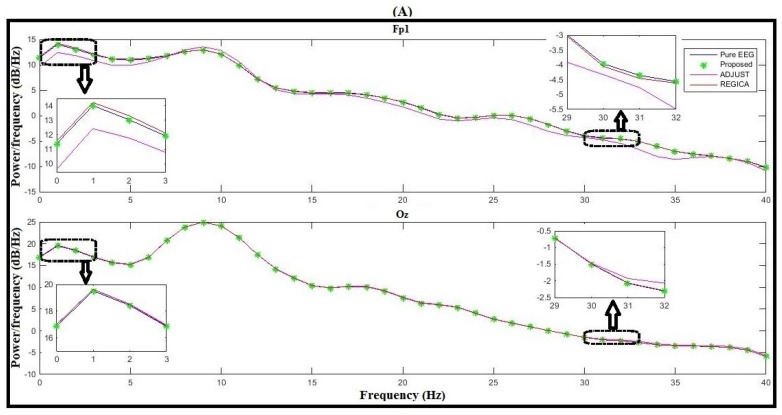

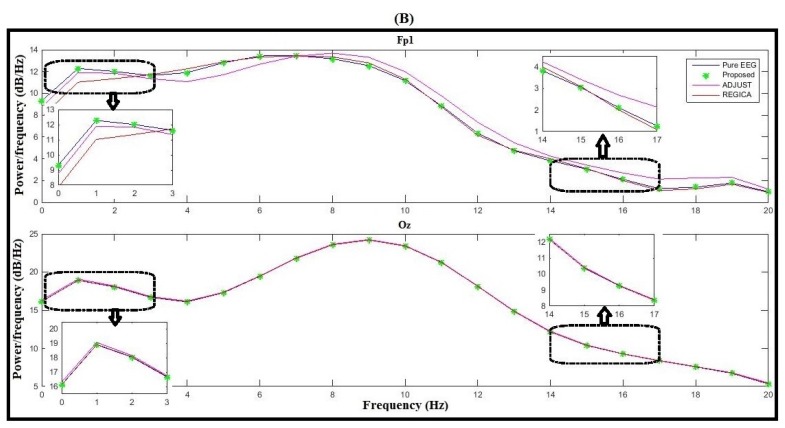
Comparison of the proposed algorithm with ADJUST and REGICA in frequency domain at Fp1 and Oz. (**A**) EEG spectra after applying filter 0.5–40 Hz; (**B**) EEG spectra after applying filter 0.5–20 Hz.

For this purpose, 10 s of artifact-free EEG data before artifact contamination is selected by an expert. Then, the PSD was computed using pwlech function in MATLAB. The resulting PSD of one subject on a frontal electrode Fp1 and a most occipital electrode Oz is shown in Figure 9. It can be seen in Figure 9A,B (upper panel) that the proposed algorithm outperforms the conventional methods (enlarged panels), whereas the PSD at Oz (bottom panel) for all the algorithms show good agreement with the PSD of the artifact-free EEG.

Furthermore, Table 2 lists the relative error indices calculated for all three algorithms with respect to the five EEG datasets. The relative error values show the superior performance of the proposed algorithm over ADJUST and REGICA for all of the datasets. A paired *t*-test was run to determine if the relative error values differed statistically among the proposed algorithm, ADJUST and REGICA. It can be seen from Table 2 that the proposed algorithm is highly statistically significant when compared with ADJUST and REGICA, except for one subject against REGICA (*p* < 0.17). For the purpose of further validation, mutual information index is used to evaluate the performance of the proposed algorithm against ADJUST and REGICA. Average mutual information values of all subjects for all electrodes are listed in Table 3. The results of mutual information index show that the proposed algorithm preserved more mutual information between the artifact-free EEG signal and the reconstructed EEG signal as compared to the conventional methods.

**Figure 10:**
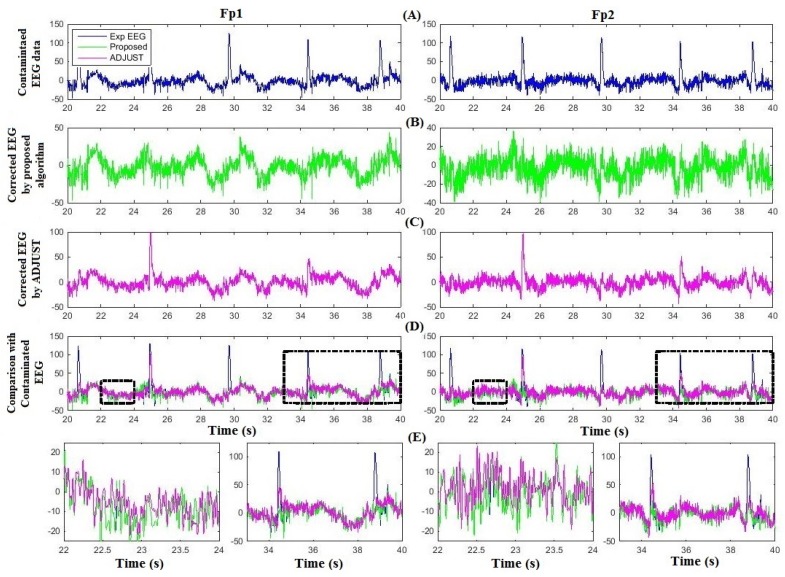
Comparison of the proposed algorithm with ADJUST using standard data. (**A**) Contaminated experimental EEG data at Fp1 and Fp2. (**B**) Corrected EEG by the proposed algorithm. (**C**) Corrected EEG by ADJUST. (**D**) Comparison of Corrected EEG with contaminated experimental EEG. (**E**) Partial enlargement of highlighted regions.

Finally, the proposed algorithm was tested on a standard EEG dataset to determine its utility for removal of ocular artifacts from EEG. Since the standard dataset does not contain EOG signals, so we only used this dataset to compare the proposed algorithm with ADJUST. Figure 10 compares the proposed method with ADJUST for artifact removal from standard EEG data at Fp1 and Fp2. Figure 10A shows the standard EEG data with the ocular contamination, Figure 10B,C, the artifact-free EEG after implementation of the proposed algorithm and ADJUST, respectively, and Figure 10D, a comparison of the proposed algorithm and ADJUST with contaminated standard EEG data. The black boxes in Figure 10D highlight the uncontaminated and contaminated EEG-signal regions, which are partially enlarged in Figure 10E. It can be visualized that the performance of the proposed algorithm in removing ocular artifacts and maintaining the neuronal-activity-related EEG signal intact is significantly better than ADJUST (Figure 10E).

## 5. Discussion

The analysis of EEG signal always requires the identification and removal of artifacts due to eye movements and blinking. In this study, a novel algorithm, based on the combination of ICA and auto-regressive exogenous model, is proposed for automatic identification and removal of ocular activities from EEG. Since using EOG is not plausible for applications like BCI, therefore in the proposed algorithm eye tracker and frontal EEG electrodes are used to remove ocular artifacts from EEG signal. In the literature, regression based techniques are the most commonly used methods to remove ocular artifacts. Although these techniques are simple and fast but due to bidirectional contamination they proved to be less effective [29]. To overcome this issue, the proposed algorithm reconstruct ICs instead of applying regression to EEG signal. Our assumption lies with the fact that the amount of the cerebral activity included in the contaminated ICs is much lower compared to that existing in the contaminated EEG signals. So, as long as the cerebral and ocular activities are derived from independent sources, the cerebral activity included in the artifactual ICs tends to be minimal. It can, therefore, be assumed that the artifactual components contain less cerebral activity common to EOG/frontal EEG. In this way, filtering artifactual ICs with auto-regressive exogenous model will cause less removal of cerebral activity. Thus, the corrected EEG contains more cerebral information in contrast to the conventional regression analysis.

Recent efforts on artifact removal in EEG signal has shown a great utility of ICA. Although the success of ICA is encouraging, it should be treated with care [49]. Existing studies have focused almost extensively on the important reduction of the typical artifacts in ICA corrected EEG signal [50,51,52], while distortion of the cerebral part of EEG signal introduced by the method as a side effect have been left unattended [35]. First, the effectiveness of ICA strongly depends on the quality of the signal decomposition. Not all signal sources may be isolated into separate components and there are no definite means to evaluate whether or not contributions of other sources confound a particular component [33]. Additionally, selection of ocular artifacts related components is another issue in ICA based methodologies. Usually this can be done by inspecting the time series and topographic maps of the ICs [31,53,54], thus relying on the subjective judgment of the experimenter. Usually, this approach leads to misclassification of ICs and divergent results. To overcome these issues, we proposed a procedure to identify eye artifact-related ICs, by composite multi-scale entropy and comparing their activations during saccade and during fixation intervals, as defined by high temporal resolution eye tracking. The identified ICs are then filtered with median absolute deviation and auto-regressive exogenous model, so that the underlying neuronal activity will be preserved and distortion in the cerebral part of EEG will be minimized. Furthermore, several BCIs have been proposed in literature by using hybrid EEG- eye tracker system. Thus, the proposed algorithm can also be used with those algorithm to improve EEG signal quality which can further be used to improve classification accuracy of the BCI. Our results suggest that the proposed algorithm outperforms ICA (Figure 5, Figure 7 and Figure 9, Table 2, Table 3, Table 4 and Table 5). It can be seen in Figure 7 and Figure 8 that artifact-free EEG by the proposed algorithm for Fp1 and Fp2 are slightly different even the input EEG is same. This might be due to the ICA decomposition of the EEG signal, since the weight matrix contain different weights for different electrodes.

Urigüen and Garcia-Zapirain [55] reviewed all previous methods for removing/reducing artifacts from EEG signal and they concluded that an optimal method should consist of combining various algorithms in cascade to enhance the quality of the signal by using multiple processing stages. The idea of recovering neuronal signal from artifactual ICs was firstly proposed by Castellanos and Makarov [35]. They proposed that artifactual ICs should not be simply replaced with zero as they might have leaked neural signal in it. Recently another methodology based on the same assumption was developed by Klados and colleagues [29]. In their method, they used regression based removal of ocular activity from ICs. Although these methods proved to be effective in removing ocular artifacts from EEG signal but they require the processing of all ICs and more computational cost which is not plausible for applications like BCI. Furthermore, processing of all ICs may cause to produce distortion in those ICs which are not related to ocular artifacts and results in distortion to EEG signal. In contrast to all these methodologies, the proposed algorithm filters only the automatically identified artifactual components using median absolute deviation and auto-regressive exogenous model. In this way more neuronal activity related information can be preserved and it requires less computational cost. Results enhance our hypothesis that instead of processing all ICs only artifactual ICs should be processed (Figure 6, Figure 8 and Figure 9, Table 2, Table 3, Table 4 and Table 5).

The performance of the proposed algorithm is compared with two state-of-the-art techniques, (1) ADJUST based ICA [34] and (2) REGICA [29]. Both conventional algorithms have been implemented through EEGLAB toolbox. In the current study, composite multi-scale entropy and eye tracker based criteria are proposed for use in automatic identification of blink and eye movement related artifactual components. To verify that the proposed algorithm can differentiate between artifactual ICs and neuronal-activity related ICs, a comparison with manual detection by two experienced experts and ADJUST was carried out. The criteria used to recognize artifactual components by EEG experts was in view of time course, topographic maps and the power spectrum plots of the ICs in EEGLAB. Notably, both experts’ selection of eye movement and blink related ICs was identical. The results of this comparison was listed in Table 4. The performance of the proposed algorithm is also statistically analyzed by calculating True positive (IC marked as artifactual both by the algorithm and visual inspection), False Positive (IC marked by the algorithm but not with the visual inspection), True Negative (IC marked neither by the algorithm nor with the visual inspection), and False Negative (IC not marked with the algorithm but with the visual inspection). The total count for each parameter for all subjects is tabulated in Table 5. Average sensitivity and average specificity for all subjects is calculated as follows [27]:

(24) $$Sensitivity=\frac{TP}{TP+FN}\times 100\%$$

(25) $$Specificity=\frac{TN}{TN+FP}\times 100\%$$

The agreement rate between visual inspection and the proposed algorithm and ADJUST was calculated using [27]:

(26) $$Agreement\;Rate=\frac{TP+TN}{TP+TN+FP+FN}$$

The agreement rate between the proposed algorithm and visual inspection is found to be 98.75%, whereas for ADJUST the agreement rate is found to be 96.25%. The result of this analysis suggest that the proposed algorithm can be used as a valuable tool for automatic identification of artrifactual ICs. Relative error is a measure which depicts the performance of each algorithm in both removing ocular artifacts as well as quantifying the amount of distortion introduced in the time-domain. Our results suggest that the proposed algorithm (0.0147 ± 0.0220) has a better performance in removing ocular artifacts from EEG signal, since it successfully removes ocular artifacts, while at the same time keeping the cerebral signal intact in the time domain when it was compared to ADJUST (0.1606 ± 0.1498) and REGICA (0.0512 ± 0.0637) algorithms. Moreover, paired *t*-test enhances the dominance of the proposed algorithm as the difference with ADJUST method is highly statistically significant for all subjects (*p* < 0.001). In case of REGICA the difference is highly statistically significant with *p* < 0.001 except for subject 2 (*p* < 0.011) and subject 5 (*p* < 0.17). Furthermore, mutual information index was adopted to investigate that how much information artifact-free EEG signal shares with the reconstructed EEG signal after the implementation of different methods. The average mutual information value for all datasets using the proposed algorithm (2.6461) against ADJUST (1.7488) and REGICA (2.2578) demonstrate improved performance of the proposed method. This analysis enhances our hypothesis that ICA and regression method are less effective in removing ocular artifacts from EEG signal. Finally, the contribution of the different component of the proposed algorithm is analyzed by eliminating different component one by one. After eliminating different components, the performance of the proposed algorithm in removing artifacts and keeping the neuronal activity related EEG signal intact is decreased and the relative error is increased. In case of eliminating composite multi-scale entropy and eye tracker based criteria for detection of artifactual ICs cause an increase in the relative error (0.4219 ± 0.0889). This enhances our hypothesis that only artifactual ICs should be identified and processed for artifact correction. Hence the utility and value of composite multi-scale entropy and eye tracker based criteria as a useful tool for identification of eye movement and blink related ICs. Furthermore, the ability of removing ocular artifacts by the proposed algorithm is decreased by the elimination of median absolute deviation. However, the elimination of median absolute deviation has very less effect on the neuronal activity related EEG signals in nonartifactual zone because median absolute deviation is only used to remove high magnitude ocular activities. Lastly, in the proposed algorithm auto-regressive exogenous and affine projection algorithm is used to remove remaining artifacts and to compensate for the possible neuronal activity loss due to median absolute deviation filtration. The elimination of auto-regressive exogenous and affine projection algorithm cause an increase in relative error (0.0253 ± 0.0156). This analysis show that the performance of the proposed algorithm is decreased by the elimination of different components. One striking feature of the proposed algorithm is that it does not require any calibration or pre-training. Also, this is one of the few studies concerning an artifact removal technique, in which statistical analysis is used to evaluate the performance of the proposed methodology.

## 6. Conclusions

This paper presents a novel algorithm using hybrid EEG and eye tracker system to automatically identify and remove ocular artifacts from EEG data by combining ICA and auto-regressive exogenous model. The performance of the proposed algorithm is demonstrated using experimental and standard EEG datasets. The proposed methodology enables the removal of ocular artifacts in the artifactual zone while keeping the neuronal activity related EEG signal intact in the non-artifactual zone. Additionally, results show that the proposed algorithm outperformed the two state of the art techniques based on ADJUST and REGICA. Relative error and mutual information are used as evaluation indexes to quantify the amount of distortion produced in corrected EEG by each algorithm. The statistical significance of the proposed algorithm is verified using paired *t*-test.

## Figures and Tables

**Table 1:** Summary of the proposed algorithm.

Input: Contaminated EEG data, Eye tracker data
Output: Artifact-free EEG data
Synchronization of EEG and eye tracker Decompose contaminated EEG data using ICA to get ICs Portioning of ICs into saccade and fixation epochs Calculate composite multi-scale entropy and ratio of mean variance to identify ocular artifacts related ICs Apply median absolute deviation to remove high magnitude ocular activities from identified ICs Filter ICs with auto-regressive exogenous model and affine projection algorithm Artifact-free EEG data by back projecting all ICs using inverse ICA

**Table 2:** Relative error values results for the proposed algorithm against ADJUST and REGICA using artifact-free EEG data.

Subject	Proposed	ADJUST ICA	*p*-val	REGICA	*p*-val
1	0.0001 ± 0.0001	0.0645 ± 0.0944	<0.001	0.0452 ± 0.0613	<0.001
2	0.0185 ± 0.0305	0.1741 ± 0.1417	<0.001	0.0437 ± 0.0393	<0.011
3	0.0146 ± 0.0230	0.1025 ± 0.1191	<0.001	0.0527 ± 0.06–	<0.005
4	0.0134 ± 0.0244	0.1893 ± 0.1582	<0.001	0.0779 ± 0.1140	<0.005
5	0.0273 ± 0.0320	0.2727 ± 0.2358	<0.001	0.0366 ± 0.0389	<0.17
Average	0.0147 ± 0.0220	0.1606 ± 0.1498		0.0512 ± 0.0637	

**Table 3:** Average mutual information values results of all subjects for the proposed algorithm against ADJUST and REGICA for all electrodes.

Electrode Location	Proposed	ADJUST	REGICA
Fp1	2.7148	0.6680	1.4023
Fp2	2.6286	0.7578	1.4563
F7	2.7169	1.1796	1.9115
F3	2.7165	1.2789	2.0176
Fz	2.6973	1.3932	1.9717
F2	2.6434	1.3789	2.0190
F8	2.5538	1.4237	1.8863
FC5	2.6946	1.2919	2.3216
FC3	2.5262	1.5692	2.3181
FC2	2.5720	1.8742	2.3239
FC6	2.6034	1.8046	2.1482
T7	2.7158	1.4571	2.3656
C3	2.6685	1.4754	2.3942
Cz	2.7435	2.0669	2.4309
C4	2.6075	1.9813	2.3062
T8	2.6611	1.8524	2.3069
TP9	2.6515	1.5851	2.3234
CP5	2.6888	1.5566	2.4402
CP1	2.6669	2.0910	2.4864
CP2	2.7186	2.1592	2.4384
CP6	2.5839	2.0236	2.3601
TP10	2.6307	1.9541	2.3272
P7	2.5600	1.8588	2.4463
P3	2.6275	2.1776	2.4715
Pz	2.6076	2.1811	2.3613
P2	2.6230	2.1739	2.3948
P8	2.6290	2.0796	2.4958
PO9	2.6540	2.2194	2.4626
O1	2.6597	2.1380	2.4375
Oz	2.6758	2.2584	2.4655
O2	2.5836	1.9191	2.3257
PO10	2.6514	2.1337	2.4338
Average	2.6461	1.7488	2.2578

**Table 4:** Comparison results for identification of artifactual ICs.

Subject	EEG Experts		Propsoed Algorithm		ADJUST	
	Vertical and Horizontal	Blink	Vertical and Horizontal	Blink	Vertical and Horizontal	Blink
1	1	1	1	1	0	1
2	1	1	1	2	0	2
3	1	2	2	2	2	2
4	2	1	2	1	2	1
5	1	1	1	1	3	1

**Table 5:** Performance evaluation of the proposed algorithm and ADJUST for all subjects.

Method	True Positive (TP)	False Positive (FP)	True Negative (TN)	False Negative (FN)	Average Sensitivity	Average Specificity
Proposed	12	2	146	0	100%	98.64%
ADJUST	10	4	144	2	83.33%	97.29%
